# 4G/5G polymorphism of *PAI-1 *gene is associated with multiple organ dysfunction and septic shock in pneumonia induced severe sepsis: prospective, observational, genetic study

**DOI:** 10.1186/cc8992

**Published:** 2010-04-29

**Authors:** Krisztina Madách, István Aladzsity, Ágnes Szilágyi, George Fust, János Gál, István Pénzes, Zoltán Prohászka

**Affiliations:** 1 Department of Anesthesiology and Intensive Therapy, Semmelweis University, Kútvölgyi út 4, Budapest, H-1125, Hungary; 2 3rd Department of Internal Medicine, Research Laboratory, Semmelweis University, Kútvölgyi út 4, Budapest, H-1125, Hungary; 3 Research Group of Inflammation Biology and Immunogenomics, Semmelweis University and Hungarian Academy of Sciences, Nagyvárad tér 4, Budapest H-1089, Hungary

## Abstract

**Introduction:**

Activation of inflammation and coagulation are closely related and mutually interdependent in sepsis. The acute-phase protein, plasminogen activator inhibitor-1 (PAI-1) is a key element in the inhibition of fibrinolysis. Elevated levels of PAI-1 have been related to worse outcome in pneumonia. We aimed to evaluate the effect of functionally relevant 4G/5G polymorphism of *PAI-1 *gene in pneumonia induced sepsis.

**Methods:**

We enrolled 208 Caucasian patients with severe sepsis due to pneumonia admitted to an intensive care unit (ICU). Patients were followed up until ICU discharge or death. Clinical data were collected prospectively and the *PAI*-1 4G/5G polymorphism was genotyped by polymerase chain reaction-restriction fragment length polymorphism technique. Patients were stratified according to the occurrence of multiple organ dysfunction syndrome, septic shock or death.

**Results:**

We found that carriers of the *PAI-1 *4G/4G and 4G/5G genotypes have a 2.74-fold higher risk for multiple organ dysfunction syndrome (odds ratio [OR] 95% confidence interval [CI] = 1.335 - 5.604; *p *= 0.006) and a 2.57-fold higher risk for septic shock (OR 95%CI = 1.180 - 5.615; *p *= 0.018) than 5G/5G carriers. The multivariate logistic regression analysis adjusted for independent predictors, such as age, nosocomial pneumonia and positive microbiological culture also supported that carriers of the 4G allele have a higher prevalence of multiple organ dysfunction syndrome (adjusted odds ratio [aOR] = 2.957; 95%CI = 1.306 -6.698; *p *= 0.009) and septic shock (aOR = 2.603; 95%CI = 1.137 - 5.959; *p *= 0.024). However, genotype and allele analyses have not shown any significant difference regarding mortality in models non-adjusted or adjusted for acute physiology and chronic health evaluation (APACHE) II. Patients bearing the 4G allele had higher disseminated intravascular coagulation (DIC) score at admission (*p *= 0.007) than 5G/5G carriers. Moreover, in 4G allele carriers the length of ICU stay of non-survivors was longer (*p *= 0.091), fewer ventilation-free days (*p *= 0.008) and days without septic shock (*p *= 0.095) were observed during the first 28 days.

**Conclusions:**

In Caucasian patients with severe sepsis due to pneumonia carriers of the 4G allele of *PAI-1 *polymorphism have higher risk for multiple organ dysfunction syndrome and septic shock and in agreement they showed more fulminant disease progression based on continuous clinical variables.

## Introduction

Sepsis is a complex clinical syndrome that results from an infection-triggered systemic inflammatory response. Despite significant advances in supportive care and in research on its pathogenesis, sepsis remains the leading cause of death in critically ill patients [1].

Patients with apparently similar general condition and severity of infection may present profoundly different survival rates. Individual differences in disease manifestation are influenced by the genetic predisposition of the patient, as recognized by the PIRO (predisposition, infection, response, organ dysfunction) concept [2]. Single nucleotide polymorphisms (SNPs) in genes involved in the inflammatory response that influence sepsis susceptibility or severity may explain the clinical variability observed during the course of similar infections.

It is already known that activation of inflammation and coagulation are closely related and mutually interdependent in sepsis [3]. The imbalance between fibrin generation and dissolution contributes to disseminated intravascular coagulation and multiple organ dysfunction syndrome (MODS) [4].

The glycoprotein serine protease plasminogen activator inhibitor-1 (PAI-1) is a key element in the inhibition of fibrinolysis. The primary role of PAI-1 *in vivo *is fast acting inhibition of tissue- and urokinase-type plasminogen activators. PAI-1 is also an acute-phase protein during acute inflammation. Plasma levels of PAI-1 are influenced by genetic, metabolic, endocrine, dietary, and physical activity factors, and they strongly increase in response to inflammation and injury [5-10]. The alveolar compartment is an important site of PAI-1 production and activity. Several studies demonstrated worse outcomes in patients hospitalized due to acute lung injury, acute respiratory distress syndrome and severe pneumonia who had increased levels of PAI-1 in bronchoalveolar lavage fluid and plasma [11,12]. In patients with sepsis, the levels of PAI-1 are positively related to poor outcome, increased severity of the disease, and increased levels of various cytokines, acute-phase proteins, and coagulation parameters [13].

The gene coding for PAI-1 has several polymorphic loci among which the most studied is the 4G/5G insertion/deletion polymorphism (rs1799768) containing either four or five (4G/5G) guanine bases at -675 within the promoter region of the human *PAI-1 *(*SERPINE1*) gene [14]. Both alleles of this SNP can bind a transcriptional activator, whereas the 5G allele binds a repressor protein at an overlapping site. Therefore homozygosity for the 4G allele renders this negative regulator unable to act, resulting in greater transcription of the *PAI-1 *gene, while heterozygotes show intermediate phenotype [4,15]. The 4G allele of the 4G/5G polymorphism has been associated with increased susceptibility to community-acquired pneumonia, and increased mortality in hospitalized patients with severe pneumonia [16,17]. In addition, the 4G allele was reported to affect the risk of developing severe complications and higher mortality in meningococcal sepsis and trauma [18-22]. Based on the above mentioned studies, we hypothesized that the carriers of the 4G allele of *PAI-1 *polymorphism have higher risk for worse outcome in pneumonia-induced sepsis.

While evaluating the effects of SNPs on individual differences in the manifestation of sepsis, clinical factors, such as the etiology of the infectious process, the virulence of the pathogenic microorganism, undrainable surgical source of sepsis, the time to hospital admission and adequate treatment, the presence of comorbidities, and differences in racial origin and gender distribution, clearly act as confounding agents. Aimed at minimizing these confounding agents - frequently not taken into consideration in previous studies - we evaluated the effect of the 4G/5G polymorphism of the *PAI-1 *gene on the occurrence of organ dysfunction, severity of the disease and mortality in a relatively homogenous cohort of patients: only Caucasian subjects with severe sepsis due to pneumonia were included in the study.

## Materials and methods

### Patients and definitions

From an original cohort of 301 critically ill patients diagnosed with sepsis consecutively admitted to the Department of Anesthesiology and Intensive Therapy of Semmelweis University, 208 patients met the criteria of severe sepsis due to pneumonia and were enrolled in the study within 24 hours of admission to the ICU. The study enrolment was carried out between June 2004 and June 2007. Exclusion criteria were: primary site of infection other than lungs, undrainable surgical source of sepsis, malignancy and final stage of chronic disease, chronic treatment with steroids or immunosuppressive drugs, AIDS and pregnancy. Patients were treated according to the Surviving Sepsis Campaign guidelines for the management of severe sepsis and septic shock [23]. Patients received empiric broad-spectrum antibiotic therapy according to the expected susceptibility of the probable pathogen. After receiving positive results (lower respiratory tract or blood culture) we de-escalated antibiotic therapy according to susceptibility of the pathogens. All patients were followed up during their hospital stay until they were discharged from the ICU or died. MODS, septic shock and death of any cause were registered as end-points of the study. Continuous variables characterising severity of illness progression such as ICU length of stay, invasive ventilation-free days and days without septic shock during the first 28 days were also evaluated.

The diagnosis of pneumonia was made on the basis of appearance of new infiltrate on the chest x-ray in the presence of cough or fever. All patients met the criteria of the British Thoracic Society for severe pneumonia [24]. MODS has been defined as "the presence of altered organ function in an acutely ill patient such that homeostasis cannot be maintained without intervention" [25]. Clinically, MODS was considered as a sequential or concomitant occurrence of a significant derangement of function in two or more organ systems of the body, against a background of critical illness. Severe sepsis was defined as acute organ dysfunction secondary to infection, and septic shock defined as severe sepsis resulting in hypotension despite adequate fluid resuscitation according to the 2001 SCCM/ESICM/ACCP/ATS/SIS International Sepsis Definitions Conference [26]. Sepsis-induced hypotension was defined as systolic blood pressure of less than 90 mmHg, mean arterial pressure of less than 60 mmHg or a systolic blood pressure decrease of more than 40 mmHg in the absence of other cause of hypotension. Disseminated intravascular coagulation (DIC) score was calculated at admission according to the International Society on Thrombosis and Haemostasis [27].

Written informed consent was obtained from patients or their relatives, and the study was approved by the local ethics committee.

Genomic DNA was extracted from white blood cells using the method described by Miller and colleagues [28]. The *PAI-1 *-675 locus was amplified using the forward 5'-CACAGAGA GAGTCTGGCCACGT-3' and the reverse 5'-CCAACAGAGGACTCTTGGTCT-3' primers. The amplified DNA was incubated with *Bsl*I restriction enzyme and the cleaved fragments were analyzed by electrophoresis in a 2% gel with ethidium bromide [29].

Data were collected in MS Excel 2003 (Microsoft, Redmond, WA, USA) and were analyzed with SPSS 13.0 for Windows (SPSS, Chicago, IL, USA) software. Categorical variables were reported as absolute numbers and percentages, and continuous variables as medians and interquartile ranges. Categorical data were compared using a Pearson Chi-squared test; continuous data were compared with categorical data using nonparametric Mann-Whitney *U *and Kruskal-Wallis tests. All reported *p *values were two-tailed and *p *< 0.05 was considered to be significant. Hardy-Weinberg equilibrium analysis was performed by comparing the detected genotype distribution with the theoretical distribution estimated on the basis of the allele frequencies.

Multiple logistic regression analysis was used to evaluate independent predictors (*p *< 0.05) for the three end-points. Hazard risk of in ICU mortality associated with genotypes and other independent variables was estimated using a Cox proportional hazards regression analysis.

*Post-hoc *power analysis was performed by Statistica software (Tulsa, OK, USA) for chi-squared test. With our sample sizes (the numbers of patients in the subgroups with MODS, shock and non-survivors were 121, 89 and 78, respectively) the minimal genotype frequency difference to be detected with a power of 0.8, were 0.145, 0.135 and 0.132, respectively.

## Results

### Patient characteristics

Of the 208 enrolled patients one was excluded due to insufficient DNA quality for genotype determination. The median age of the 207 septic patients was 65 (53 to 75) years and their gender distribution was 50.2% males and 49.8% females.

All patients had at least one organ dysfunction (respiratory insufficiency) and 121 (58.5%) had MODS. Eighty nine (43%) subjects met the criteria for septic shock and 78 (37.7%) patients died during the ICU stay. The cause of deaths was cardiovascular collapse due to MODS. One hundred and sixty-three (78.7%) patients required invasive and 44 (21.3%) required non-invasive ventilation during the ICU stay. Pathogen microorganism could not be confirmed in 86 (41.5%) cases.

Analyzing the differences among cohorts stratified by the three end-points, we found that the median age was significantly higher in patient with worse outcomes (MODS, septic shock and non-surviving patients; Table 1). There was a tendency toward lower mortality in women than in men (*p *= 0.051). The incidence of comorbid conditions did not differ in the three end-points, except for pulmonary hospitalization in the two years preceding current ICU stay, which was, interestingly, less frequent in the septic shock cohort than in severe sepsis and nosocomial infection, which was significantly more prevalent in patients with MODS and septic shock as compared with the group of non-MODS and severe sepsis, respectively. As expected, patients with worse outcomes had higher Acute Physiology and Chronic Health Evaluation II (APACHE II) and DIC scores at admission. Occurrence of MODS (*p *< 0.001) and septic shock (*p *= 0.011) was significantly more prevalent in patients with positive culture and targeted antibiotic treatment than in unconfirmed cases with empiric treatment.

**Table 1:** Summary of patient characteristics stratified by multiple organ dysfunction, sepsis severity and mortality

	Multiple organ dysfunction		Sepsis severity		ICU mortality	
	
Patient characteristics Median (quartiles) or %	Non-MODS (n = 86)	MODS (n = 121)	Severe sepsis (n = 118)	Septic shock (n = 89)	Survivors (n = 129)	Non-survivors (n = 78)
**Demographics**						
Age (years)	58.5 (48-68)*	70 (59-77)*	61 (51-71)*	72 (59-77)*	61 (50-70)*	72.5(61-79)*
Male sex, %	45.3	53.7	46.6	55.1	45.0	59.0
**Previous or preexisting conditions**						
Days of complaint before admission, (days)	4 (2-7)	4 (1-10)	4 (2-8)	4 (1-9)	4 (1-7.5)	4 (2-10)
Pulmonary hospitalisation in the past two years, %	27.9	24.8	31.4*	19.1*	27.9	23.1
Cachexia, %	12.8	17.4	12.7	19.1	14.0	17.9
Nosocomial pneumonia, %	25.6*	44.6*	30.5*	44.9*	32.6	43.6
Ischemic heart disease, %	33.7	43.8	37.3	42.7	36.4	44.9
COPD, %	43.0	33.1	42.4	30.3	39.5	33.3
Hypertension, %	55.8	62.8	59.3	60.7	58.1	62.8
Diabetes mellitus, %	24.4	27.3	26.3	25.8	24.0	29.5
Active and ever (>15 years) smoking, %	46.5	42.1	46.6	40.4	46.5	39.7
Alcohol consumption >1 glass/day, %	9.3	13.2	9.3	14.6	10.9	12.8
**ICU conditions**						
28 days mortality, %	0.0*	62.8*	9.3*	73.0*	0.0*	97.4*
ICU mortality, %	0.0*	64.5*	9.3*	75.3*	-	-
APACHE II score at admission	18 (14-22)*	24 (18.5-30.5)*	19 (15-23)*	25 (20-31)*	19 (15-23)*	25 (22-31)*
DIC score at admission	0 (0-2)*	3 (0-4)*	0 (0-2)*	3 (2-5)*	0 (0-2)*	3 (2-4)*
ICU length of stay, (days)	8 (5-12)	8 (5-16.5)	8 (5-12)	8 (4.5-16.5)	9 (5-15)	8 (4-12)
Horowitz quotient of invasively ventilated patients, (PaO_2_/FiO_2_)	269.0 (161.3-342.3)*	179.6 (126.2-232.4)*	222.0 (149.9-335.4)*	175.2 (119.7-229.7)*	197.5 (139.1-305.5)	188.3 (124.2-241.6)
ARDS, %	7.0*	24.0*	8.5*	28.1*	11.6*	25.6*
Invasive ventilation, %	52.3*	97.5*	62.7*	100.0*	65.9*	100.0*
Length of invasive ventilation, (days)	5 (3.5-7.5)*	8 (4-13.3)*	5.5 (4-10.3)	7 (4-14)	6 (4-13)	7 (4-12)
Tracheotomy, %	5.8*	14.9*	10.2	12.4	10.9	11.5
Hemodialysis, %	7.0*	17.4*	9.3	18.0	9.3*	19.2*
**Infection types **						
Unconfirmed, **%**	55.8*	31.4*	49.2*	31.5*	45.0	35.9
Confirmed, **%**						
Gram positive	10.5	19.3	11.7	21.3	15.5	18.0
Gram negative	52.6	57.8	60.0	52.5	52.1	62.0
Atypical pneumonia (Chlamydia, Mycoplasma, Legionella)	23.7	14.5	18.3	16.4	21.1	12.0
Mixed	13.2	8.4	10.0	9.8	11.3	8.0

*Statistical significance (*P *< 0.05) of comparing the groups of non-MODS vs. MODS, severe sepsis vs. septic shock and survivors vs. non-survivors by Mann-Whitney or Pearson Chi-square tests

APACHE II, acute physiology and chronic health evaluation II; ARDS, acute respiratory distress syndrome; COPD, chronic obstructive pulmonary disease; DIC, disseminated intravascular coagulation; FiO_2_, fraction of inspired oxygen; MODS, multiple organ dysfunction syndrome; PaO_2_, partial pressure of arterial oxygen.

### Genotype distribution

Distribution of the genotypes of *PAI-1 *SNP is shown in Table 2. In the studied population, the genotype frequencies were: 4G/4G = 30.4% (n = 63); 4G/5G = 50.7% (n = 105); and 5G/5G = 18.8% (n = 39). Genotype frequencies were in Hardy-Weinberg equilibrium (*p *= 0.92). The calculated allele frequency was 0.56 for 4G and 0.44 for 5G.

**Table 2:** The incidence of MODS, septic shock and non-survival in carriers of the different *PAI-1 *4G/5G genotypes

Genotype	n	MODS (%)	Septic shock (%)	Non-survivors (%)
4G/4G	63	39 (61.9%)	32 (50.8%)	27 (42.9%)
4G/5G	105	67 (63.8%)	47 (44.8%)	41 (39.0%)
5G/5G	39	15 (38.5%)	10 (25.6%)	10 (25.6%)

MODS, multiple organ dysfunction syndrome; PAI-1, plasminogen activator inhibitor 1.

### Clinical associations of *PAI-1 *4G/5G polymorphism

The incidence of MODS, septic shock and non-survival were similar and higher in carriers of 4G/4G and 4G/5G genotypes than in patients with 5G/5G genotype. Therefore, these two genotypes were combined in further analyses (Table 2.).

Genotype distribution and allele frequencies of the *PAI-1 *4G/5G polymorphism stratified by MODS, sepsis severity and ICU mortality are shown in Table 3. The 4G/4G and 4G/5G genotypes were significantly more frequent in MODS and in septic shock compared with non-MODS and severe sepsis, respectively. Consequently the risk of MODS was 2.74-fold (odds ratio (OR) = 2.74 95% confidence interval (CI) = 1.335 to 5.604; *p *= 0.006) and the risk of septic shock was 2.57-fold (OR = 2.57) 95% CI = 1.180 to 5.615; *p *= 0.018) higher in carriers of the *PAI-1 *4G/4G and 4G/5G genotypes than in individuals bearing the 5G/5G genotype. Accordingly, the frequency of *PAI-1 *4G allele in the group of MODS (OR = 1.495; 95% CI = 1.008 to 2.217; *p *= 0.045) and septic shock (OR = 1.601; 95% CI = 1.077 to 2.381; *p *= 0.019) was significantly different from that of non-MODS and severe sepsis, respectively.

**Table 3:** Distribution of *PAI-1 *4G/5G genotypes and alleles as stratified according to multiple organ dysfunction, sepsis severity and mortality

	Multiple organ dysfunction			Sepsis severity			ICU mortality		
	
	Non-MODS (n = 86)	MODS (n = 121)	*p *value*	Severe sepsis (n = 118)	Septic shock (n = 89)	*p *value*	Survivors (n = 129)	Non-survivors (n = 78)	*p *value*
**Genotype**									
4G/4G and 4G/5G	62 (72.1%)	106 (87.6%)		89 (75.4%)	79 (88.8%)		100 (77.5%)	68 (87.2%)	
5G/5G	24 (27.9%)	15 (12.4%)	0.005	29 (24.6%)	10 (11.2%)	0.015	29 (22.5%)	10 (12.8%)	0.085
									
**Allele**									
4G	86 (50.0%)	145 (59.9%)		120 (50.8%)	111 (62.4%)		136 (52.7%)	95 (60.9%)	
5G	86 (50.0%)	97 (40.1%)	0.045	116 (49.2%)	67 (37.6%)	0.019	122 (47.3%)	61 (39.1%)	0.104

*Pearson Chi-square tests

MODS, multiple organ dysfunction syndrome; PAI-1, plasminogen activator inhibitor 1.

Comparing the genotype distribution between surviving and non-surviving patients, there was a tendency towards higher frequency of the 4G/4G and 4G/5G genotypes (*p *= 0.085) in non-survivors. However, allele frequencies of the *PAI-1 *4G/5G polymorphism were not different in the same subgroups.

Analyzing the DIC score at admission among carriers of different *PAI-1 *genotypes, we found that patients bearing the 4G allele had significantly higher DIC scores at the time of admission than 5G/5G homozygotes (2 (0 to 3) vs. 0 (0 to 2), *p *< 0.007). We also evaluated the association of *PAI-1 *polymorphism with ICU length of stay, invasive ventilation-free days and days without septic shock during the first 28 days of ICU stay. The length of ICU stay did not differ between carriers and non-carriers of the 4G allele (*p *= 0.858). However, in non-survivors the median ICU length of stay was more than two days lower in patients with the 4G allele than in 5G/5G patients (6 (4 to 11) vs. 8.5 (6 to 18), *p *= 0.091). Carriers of the 4G allele had significantly less invasive ventilation free-days during the first 28 days than patients with the 5G/5G genotype (0 (0 to 0) vs. 0 (0 to 6), *p *= 0.008). The median of days without septic shock during the first 28 days was lower in patients bearing the 4G/4G and 4G/5G genotypes than in carriers of the 5G/5G genotype (4 (0 to 9) vs. 6 (5 to 9), *p *= 0.095).

### Multivariate analysis of factors associated with endpoints

By multivariate logistic regression analysis, three factors were independently associated with MODS and sepsis severity: age, incidence of nosocomial pneumonia and positive microbiological culture. Therefore, these three parameters were introduced simultaneously as adjusting variables in logistic regression models of MODS and severity. The adjusted model indicated an independent association of *PAI-I *4G/5G and 4G/4G genotypes with MODS and septic shock (Table 4).

**Table 4:** Multivariate logistic regression analysis of MODS, septic shock and ICU mortality

	MODS		Septic shock		ICU mortality	
	
	Odds ratio (95% confidence interval)	*p *value	Odds ratio (95% confidence interval)	*p *value	Odds ratio (95% confidence interval)	*p *value
Age, years	1.048 (1.027-1.069)	<0.001	1.032 (1.013-1.052)	0.001	-	-
Nosocomial infection	3.029 (1.503-6.102)	0.002	2.047 (1.104-3.797)	0.023	-	-
Positive microbiological culture	3.642 (1.876-7.069)	<0.001	2.365 (1.275-4.386)	0.006	-	-
APACHE II	-	-	-	-	1.158 (1.101-1.217)	<0.001
*PAI-1*						
5G/5G	reference group		reference group		reference group	
4G/5G and 4G/4G	2.957 (1.306-6.698)	0.009	2.603 (1.137-5.959)	0.024	1.998 (0.879-4.540)	0.098

APACHE II, acute physiology and chronic health evaluation II; MODS, multiple organ dysfunction syndrome.

A possible association between baseline variables and ICU mortality was studied by multivariate regression analysis as well. Because of multicolinearity, only the APACHE II score turned out to be independent predictor for ICU death. Therefore, this single parameter was introduced into the model. A tendency for increased risk of death was observed for the carriers of 4G/4G and 4G/5G genotypes after adjustment (Table 4). This result was confirmed by using Cox regression analysis adjusted for APACHE II score as a covariate (4G/4G and 4G/5G hazard ratio = 1.866; 95% CI = 0.897 to 3.882; *p *= 0.095).

## Discussion

This study is unique in evaluating the effect of *PAI-1 *4G/5G polymorphism on MODS, sepsis severity and mortality in a relatively homogeneous cohort of patients of the same ethnicity. Moreover, this study is the first report, to our knowledge, that shows genetic association between the 4G allele of 4G/5G polymorphism and the occurrence of MODS and septic shock in pneumonia sepsis.

Comparing the allele frequencies of *PAI-1 *4G/5G polymorphism of the studied population and that of other collections of Caucasian healthy individuals (frequency of 4G allele varied in between 0.51 and 0.55), no difference could be found [18,30].

Based on the distribution of *PAI-1 *genotypes in the three endpoints of the study, the 4G/4G and 4G/5G groups were combined for further analyses. Previous studies used both the comparison of 5G carriers versus 4G/4G and 4G carriers versus 5G/5G patients [16,31]. According to the intermediate PAI-1 level in heterozygotes, both classifications could be correct [32].

Our results showed that the risk of MODS after pneumonia sepsis was almost three times higher in carriers of the *PAI-1 *4G/5G and 4G/4G genotypes than in patients bearing the 5G/5G genotype. The multivariate logistic regression analysis adjusted for gender, age and nosocomial pneumonia also supported the hypothesis that the *PAI-1 *4G/5G polymorphism was an independent predictor of MODS. A definite explanation for the development of MODS has not yet been found; accordingly several hypotheses exist for its pathogenesis. One of them is the microvascular failure hypothesis suggesting that microvascular thrombosis may be responsible for clinical MODS [33]. Plasma PAI-1 plays an important role in microvascular fibrin depositing in septic cases and therefore may contribute to MODS and decreased survival in such patients [34]. Moreover, Menges and colleagues showed an association between the *PAI-1 *4G allele and MODS in severely injured patients [21] while Garcia-Segarra and colleagues found the same effect in a cohort of septic shock patients studying sepsis of mixed origin [20].

In addition, both adjusted and non-adjusted models supported a higher risk for septic shock in carriers of the *PAI-1 *4G allele. These findings were in agreement with the study by Westendorp and colleagues who found that patients with meningococcal disease whose relatives were carriers of the 4G/4G genotype had a six-fold higher risk of developing septic shock compared with all other genotypes [22]. On the other hand, Garcia-Segarra and colleagues and Jessen and colleagues have reported that the 4G allele of the *PAI-1 *gene was not associated with septic shock in patients with mixed type of sepsis and Gram-negative sepsis [20,35].

Finally, we analyzed the effect of *PAI-1 *4G/5G polymorphism on mortality. A tendency for a higher rate of non-survival in 4G/4G and 4G/5G carriers was observed with and without adjusting for the confounding variables. Previous studies have yielded positive findings on the impact of the 4G/5G polymorphism on mortality in subjects with sepsis due to meningococcus meningitis, trauma and burn injury [18,20,21,29,30,36]. However, others were unable to demonstrate an association between this polymorphism and mortality in patients with meningococcal sepsis [22] and Gram-negative sepsis [35].

We also analyzed the correlation between *PAI-1 *genotypes and DIC score and found that carriers of the 4G allele had higher scores at admission than patients with the 5G/5G genotype verifying that this polymorphism may influence outcome of sepsis through the disturbance of coagulation. This result was in agreement with the study by Binder and colleagues who found correlation between the *PAI-1 *4G/5G genotypes and the development of DIC in patients with meningococcal infection [19].

Continuous variables characterising severity of illness progression such as ICU length of stay, invasive ventilation-free days, and days without septic shock during the first 28 days were also evaluated according to genotypes. In the case of ICU length of stay in non-survivors and of the two other variables, 4G allele carriers showed more fulminant disease progression than 5G/5G homozygotes.

The present study has a number of strengths. The prospective design helps to reduce the phenotype misclassification. We could minimize potential bias by the restriction of inclusion criteria to patients with severe sepsis due to pneumonia, with similar complaint duration until hospital admission, and with no drainable surgical source of sepsis. The homogenous racial cohort limited the confounding genetic factors caused by ethnic heterogeneity. Moreover, we have evaluated the impact of a genetic variant on MODS, sepsis severity and mortality in multivariate regression models in order to avoid the influence of independent predictors.

In spite of the above-mentioned strengths of our study, we declare some limitations. First, a larger study size would probably provide an even stronger statistical power, therefore further studies with increased numbers of patients are required to validate our findings. Second, in this work we tested only one polymorphism of the *PAI-1 *gene and the studied population consisted of relatively elderly subjects. The observed association with the 4G/5G polymorphism may be due to its linkage disequilibrium with other functional polymorphisms in the *PAI-1 *gene. In contrast, Kathiresan and colleagues identified 2 genetic variants from 18 SNPs of the *PAI-1 *gene, rs2227631 and the 4G/5G polymorphism, which were in tight linkage disequilibrium with each other and strongly associated with plasma PAI-1 level [37]. We have chosen the 4G/5G insertion/deletion polymorphism for analysis because it is a well-characterized variation of the *PAI-1 *gene that has been studied both in normal individuals and in different diseases. Third, we could not provide data on PAI-1 serum levels and the DIC score was only available at the time of admission. Follow up of these parameters may give further details on the role of PAI-1 and coagulation in disease progression of pneumonia-induced sepsis.

## Conclusions

In summary, our results indicate that among patients hospitalized with severe sepsis due to pneumonia, carriers of the *PAI-1 *4G/4G and 4G/5G genotypes have higher risk for MODS and septic shock. This observation supports previous studies reporting that the activation of coagulation and the inhibition of fibrinolysis are important in the pathogenesis of sepsis and support the notion that particular genetic factors may predispose to worse outcome in severe sepsis. Identifying these genetic factors might, in the future, help to choose the appropriate therapy for patients at different risk.

## Key messages

• Carriers of the 4G allele of *PAI-1 *polymorphism have higher risk for MODS and septic shock in Caucasian patients with severe sepsis due to pneumonia according to both adjusted and non-adjusted analyses.

• Disease progression is more fulminant in 4G allele carriers as indicated by the association of *PAI-1 *genotypes with continuous clinical variables such as ICU length of stay in non-survivors, invasive ventilation-free days, and days without septic shock during the first 28 days.

## Abbreviations

APACHE II: acute physiology and chronic health evaluation II; CI: confidence interval; DIC: disseminated intravascular coagulation; MODS: multiple organ dysfunction syndrome; OR: odds ratios; PAI-1: plasminogen activator inhibitor 1; SNP: single nucleotide polymorphism.

## Competing interests

The authors declare that they have no competing interests.

## Authors' contributions

MK and AI were the main researcher for this study and co-contributed to write this manuscript. GJ and PI were involved in the collection of blood samples and clinical data. SzA helped in the technical work. FG and PZ contributed to the experimental design. All authors read, approved and contributed to the final manuscript.
